# *Chroococcus turigidus*, a novel cyanobacterial source for l-sparaginase production

**DOI:** 10.1038/s41598-025-19839-1

**Published:** 2025-09-29

**Authors:** Reham Gamal, Esmail M. El-Fakharany, Khaled N. M. Elsayed, Amany Salama El Sharkawy, Nader Saad Elsayed, Ola Kh. Shalaby

**Affiliations:** 1 National Institute of Oceanography and Fisheries, NIOF, Cairo, Egypt; 2 Protein Research Department, Genetic Engineering and Biotechnology Research Institute (GEBRI), City of Scientific Research and Technological Applications (SRTA-City), New Borg El-Arab, Alexandria, 21934 Egypt; 3 Botany and Microbiology Department, Faculty of Science, Beni-Suef University, Beni-Suef, 62511 Egypt; 4 Soil and Agricultural Chemistry Department, Faculty of Agriculture (Saba-Basha), Alexandria University, Alexandria, Egypt; 5 College of Resources, Hunan Agricultural University, Changsha, China

**Keywords:** Anti-cancer, Asparaginase, Chroococcus turigidus, Marine microalgae, Biochemistry, Biotechnology, Cancer, Microbiology, Plant sciences

## Abstract

The anti-leukemic drug enzyme L-asparaginase is highly sought for its potential in treating various solid tumors; as well as its application in food production for mitigation of the carcinogenic acrylamide. Marine microalgae are well known for their diverse bioactive products and ease of cultivation, making them ideal candidates for large-scale production. Seven marine microalgae were isolated based on their L-asparaginase production, and the isolate *Chroococcus turigidus* was identified as the highest producer, yielding 212.413 IU/ml. The in-vitro anticancer activity of the algal extract was evaluated against breast carcinoma (MDA) and hepatoma (HepG-2) cell lines with comparison to normal human skin fibroblast (HSF) cell lines. The IC_50_ values of the algal extract against HSF cells were Determined to be 844.4 and 730.5 µg/mL after 24 h and 48 h of treatment, while low IC_50_ values of 126.3 and 169.8 µg/mL were observed with selectivity indices (SI) of 6.69 and 4.97 respectively. The results demonstrated that, HepG-2 cells exhibited slightly greater sensitivity to the treatment compared to MDA cells. These findings suggested that *Chroococcus turigidus* could serve as a novel and abundant source of L-asparaginase enzyme and is well-suited for biomass production. To the best of our knowledge, this is the first report on L-asparaginase production from *Chroococcus turigidus*.

## Introduction

Marine microalgae are categorized into three main groups: blue-green algae (Cyanobacteria), diatoms (Bacillariophyta), and dinoflagellates (Dinophyceae)^1^. In recent years, the economic importance of marine microalgae has been greatly considered for their applications, which range from simple biomass production for human nutrition and feed in aquaculture to valuable products for medical and cosmetics fields as well as clean energy (biodiesel, biogas, bioethanol)^2–6^.

Cervical cancer develops primarily from persistent high-risk human papillomavirus (HPV) infections. When HPV enters cervical cells, it disrupts their normal physiology, leading to precancerous lesions and, over time, malignant transformation^7^. It is worth mentioning that, to date, there are no drug treatments that cause a cure or total remission of lesions caused by HPV^8^. HPV 18 is among those most involved in carcinogenesis in this region, which is why the use of HeLa cells in this study is justified since they are cervical carcinoma cells transformed by HPV 18^7^.

It is known that L-asparaginase is an amidohydrolase^9^, which plays a significant role in the pharmaceutical industry, particularly in the treatment of specific cancers, due to its antitumor properties. Some studies have demonstrated its cytotoxic effect against HeLa cells^10^. This enzyme is industrially produced and commercially available, constituting 40% of global enzyme Demands, representing USD 380 million in sales in 2017. This number is predicted to increase to USD 420 million by 2025^11^.

Recently, L-asparaginase has been used in food technology as a potent mitigating agent for reducing acrylamide (AA, CH_2_ = CH-CO-NH_2_). This potential carcinogen is formed in the reaction of L-asparagine (L-AsnA) and reducing sugars contained in foods during heating processes^12^. Microorganisms are the best sources of enzyme production. The enzyme has been produced and characterized from several bacterial genera in addition to recombinant forms. Microalgae, filamentous fungi, and higher plants also produce it^13^.

In terms of yield, *Spirulina maxima* produced 51.28 IU/L of L-asparaginase under optimized nitrogen conditions in large-scale cultures, while *Oscillatoria terebriformis* achieved about 55.56 U/mL when immobilized on Luffa pulp at optimized conditions. Various fungi, including *Aspergillus sydowii* and *Fusarium oxysporum*, Demonstrated comparatively higher yields in the range of 37–124 U/mL under different cultivation conditions. In contrast, thermophilic bacteria such as *Melioribacter roseus* were primarily noted for their exceptionally high specific activities (up to 1530 U/mg protein), making their yield data not directly comparable to the reported IU/L or U/mL values^14^.

Recently, L-asparaginase production from blue-green microalgae has been receiving more attention because they have many advantages, such as high nutrient contents, low cost of production, cost-effectiveness, no seasonal variation, highly efficient producers, being easily cultured and harvested at large scales, cheaper and easier extraction, and higher yields and purification of protein and enzymes by simple methods are available. However, few reports regarding the production of enzymes by blue-green algae have been recorded^15^. Optimization of enzyme production is very important before industrial-scale production can be considered. The medium and environmental conditions of the culture lead to modifications in cellular metabolism^16,17^.

## Materials and methods

### Algal species isolation

**Fig. 1:**
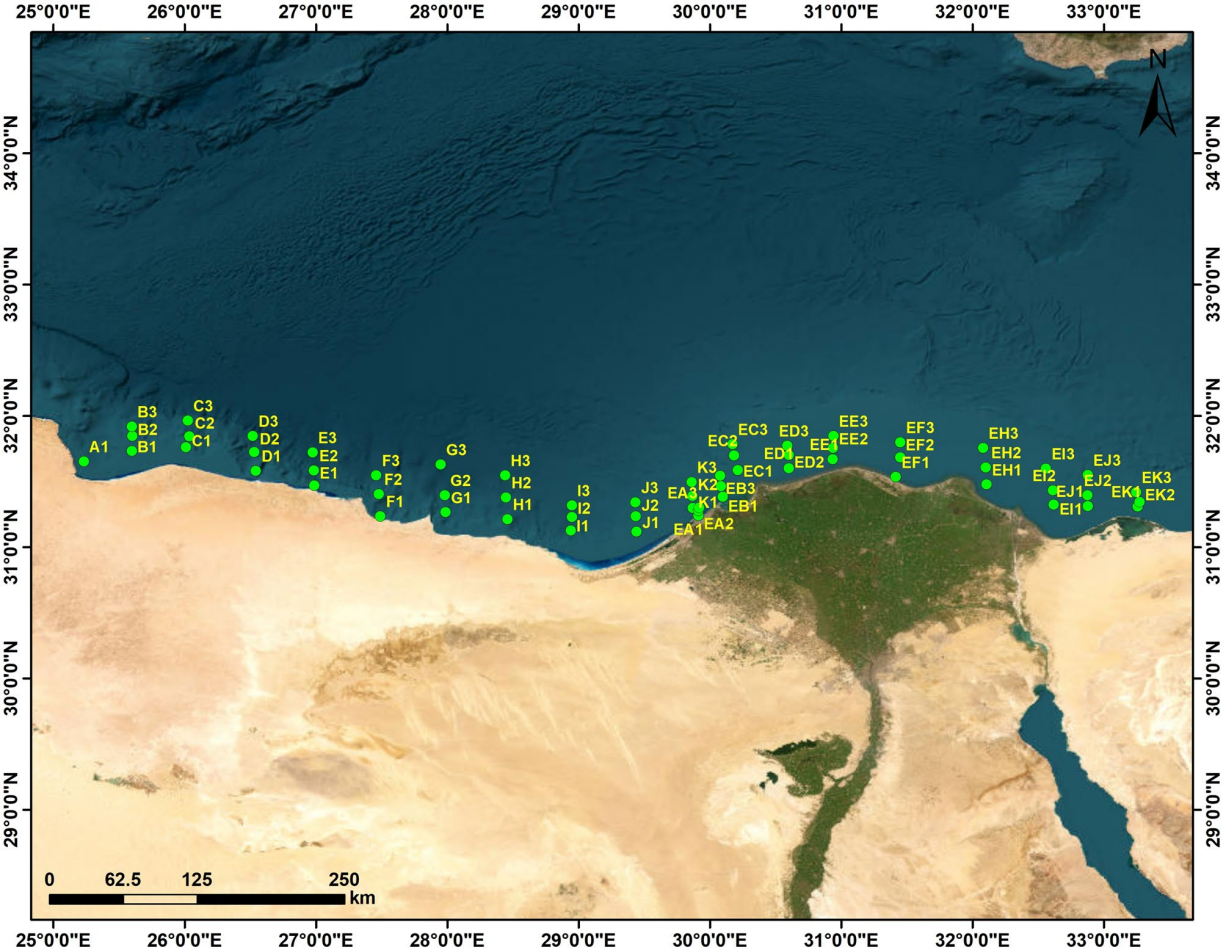
Marine water samples were collected by the EL-YARMOUK research vessel team during September 2023. Sampling sites were from the Mediterranean Sea (North of Delta, Egypt) surveying from 31° 13′ 15.6″ N 29° 53′ 06.0″ E to 31° 40′ 40.8″ N 31° 51′ 39.6″ E at 10 m depth using Google Maps. (2025). Los Angeles, CA., from https://www.google.com/maps.

Marine water samples were collected by the EL-YARMOUK research vessel team during September 2023. Sampling sites were from the Mediterranean Sea (north of Delta, Egypt), surveying from 31° 13′ 15.6" N 29° 53′ 06.0" E to 31° 40′ 40.8" N 31° 51′ 39.6" E, at 10 m depth (Fig. 1). Samples were grown in F/2 medium (Guillard et al. 1975). Cells were cultured in a 250 ml Erlenmeyer flask containing 100 ml of sterilized medium and incubated at 30 ± 2 °C for 20 days. The flask was illuminated by fluorescent white lamps at an intensity of 100 µmol photons m^−2^ s^−1^. Harvesting took place by centrifugation at 5000 rpm for 15 min at 4 °C.

### Screening for microalgae L-asparaginase production

L-asparaginase production can be determined by two techniques: qualitative methods and quantitative methods.

### Qualitative method (primary screening of L-asparaginase production)

Rapid screening of L-asparaginase produced from algal cultures was assessed using phenol red as a pH indicator in the medium^18^. Phenol red is yellow at acidic pH and turns to pink at alkaline pH; thus, a pink colour is formed by algal cultures producing L-asparaginase. Screening of potential L-asparaginase-producing algae was carried out using asparagine; pH was adjusted to 6.8 and supplemented with phenol red as a pH indicator (0.009% final concentration). Tubes were examined for change in colour of cultures from yellowish to pink due to change in pH, indicating the positive asparaginase activity, and used for further study.

### Quantitative method

The modified F/2 medium (containing phenol red as a pH indicator) served as a production medium (was adjusted to pH 7.0 before autoclaving). After sterilization, the medium (50 ml placed in 250 ml Erlenmeyer flasks) was inoculated with 5 ml of old seeded microalgae cultures and incubated at 30 °C for 20 days^19^. The L-asparaginase activity was determined by the nesslerization method.

### L-asparaginase enzyme assay

L-asparaginase enzyme activity of the isolated marine microalga was estimated by determining the amount of released ammonia derived from hydrolysis of L-asparagine by L-asparaginase according to a previously reported method using a reagent with slight modifications^20^. Briefly, the enzyme assay reaction mixture contained 900 µL of 0.05 M L-asparagine substrate, dissolved in 0.05 M Tris-HCl buffer, pH 7.5, and 100 µL of an appropriate dilution of the cell-free extract. This mixture was incubated at 37 °C for 10 min. After that, 500 µL of 15% trichloroacetic acid (TCA) was added, and the mixture was kept at room temperature for a further 10 min and centrifuged at 10,000 rpm for 10 min at room temperature. Then, 150 µl of this mixture was withdrawn and was mixed with 1400 µl of distilled water. Nessler’s reagent (200 µl) was added. After 10 min, the absorbance of the Developed colour was measured spectrophotometrically at 480 nm. Control reactions were performed under the same conditions as the enzyme assay, except that TCA was added before the enzyme sample to inactivate the reaction. One arbitrary unit of enzyme activity was Defined as the amount of enzyme that releases 1 µmole of ammonia per minute under the stated assay conditions. A standard curve using ammonium chloride was established.

$$\:Enzyme\:activity\:(IU/\text{m}\text{L})\:=\:\frac{(Abs480\times\:Vt)}{(\epsilon\:\times\:l\times\:t\times\:Vs)}$$

where Abs_480_ is the absorbance at 480 nm, VtV_tVt​ is the total reaction volume, ε is the molar extinction coefficient of ammonia (12,300 M^−1^cm^−1^), lll is the path length (1 cm), ttt is incubation time (min), and VsV_sVs​ is the sample volume.

### Anti-cancer effect

The effect of the algal extract on the viability of normal and cancer cell lines was examined using the colorimetric MTT (3-(4, 5-dimethylthiazol-2-yl)−2, 5-diphenyltetrazolium bromide) assay (Orabi et al., 2020). HSF (somatic cells obtained from normal human skin tissue), HepG-2 (hepatoma), HepG-2 (hepatoma cells derived from human liver tissue), and MDA (breast cancer) cell lines (1.0 × 104) were seeded in four 96-well tissue culture sterile microplates at 37 °C for 24 h in a 5% CO₂ incubator. HSF and MDA cells were cultured in DMEM media (SERANA, Germany); however, HepG-2 cells were cultured in RPMI-1640 media (Elabscience, China) supplemented with 10% fetal bovine serum (FBS, Gibco, USA). The cells were exposed to the algal extract at different concentrations (12.5, 25, 50, 100, 200, and 400 µg/mL) and incubated at 37 °C in a 5% CO₂ incubator. After incubation for 24 and 48 h, the cells were washed three times with PBS to remove the exposed extract, debris, and dead cells. Then, MTT solution (0.5 mg/mL) was added to the cells and incubated at 37 °C for 3 h. The formed formazan crystals were dissolved in dimethyl sulfoxide (DMSO), and absorbance was read using a microplate ELISA reader at 570 nm. All experiments were done in triplicates; the data were graphically depicted as viability versus concentrations. The half-maximal inhibitory concentration (IC50) value of the algal extract was calculated using the GraphPad Prism 8.0 software. Furthermore, the selectivity index (SI) value of the algal extract was estimated by dividing the IC50 value of normal HSF cells by the IC50 value of the treated cancer cells (Habib et al., 2022; Ebraheem et al., 2024). In addition, the effect of the algal extract on the morphology of the treated cells was investigated at different doses (50, 100, and 200 µg/mL) and visualized under ZOE fluorescence phase-contrast microscopy (BIO RAD, Singapore) and compared to untreated cells as reference cells.

All cell lines used (HSF, HepG-2, and MDA) were obtained from the American Type Culture Collection (ATCC). Experiments were performed following institutional biosafety guidelines approved by the National Institute of Oceanography and Fisheries, Egypt.

### Nuclear staining analysis

The apoptotic effect of algal extract on HepG-2 cells was studied using propidium iodide (PI) as a single nuclear staining dye and ethidium bromide/acridine orange (EB/AO) as double nuclear staining dyes. After culturing HepG-2 cells overnight, cells were exposed to the algal extract at concentrations of 50, 100, and 200 µg/mL for 48 h. The apoptotic effect was examined, and microphotographs were captured using ZOE fluorescent microscopy (BIO RAD, Singapore), as previously reported^21^.

### Migration Inhibition assay

HepG-2 cells were cultivated in a 12-well tissue culture plate until 90% confluence and then examined for migration inhibition using a wound healing assay. A sterile tip was used to scratch HepG-2 cells before treatment with the algal extract at concentrations of 50, 100, and 200 µg/mL. The wound closure area was determined and captured by imaging analysis CellSens software v1.16 after treatment for 24 h. The wound gap area measurements at zero time and treatments were determined, and the inhibitory migration factors were represented by the gap area value of treated cells over the initial scratch area^22^.

### Statistical analysis

All measurements were carried out in triplicate. Statistical analyses were performed using one-way analysis of variance (ANOVA), and the significance of the difference between means was determined by Duncan’s multiple range tests. Differences at *p* < 0.05 were considered statistically significant. The results were presented as mean values (± SD, standard deviations).

## Results

### Screening for isolates with the highest L-asparaginase production

**Fig. 2:**
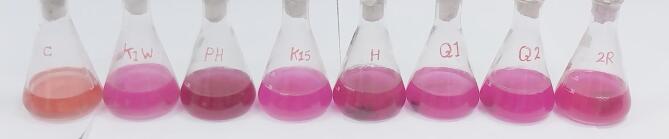
The microalgal isolates were allowed to grow on a modified F/2 medium (supplemented with phenol red as an indicator) at a temperature of 30 °C and pH 8.

**Fig. 3:**
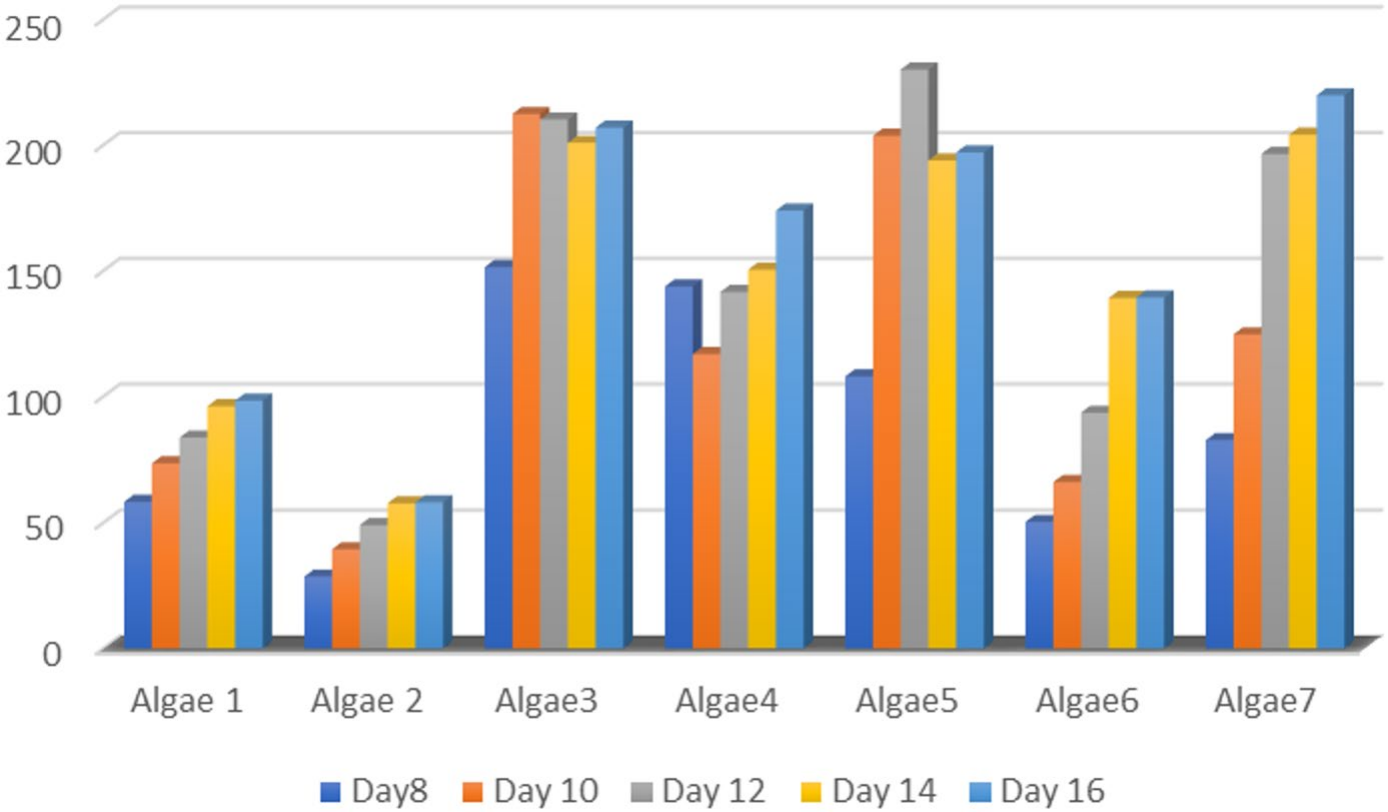
Seven microalgae (*Oscillatoria nigroriridus (1)*, *Anabaena sp.(2)*, *Chroococcus turigidus (3)*, *Oscilatoria acitissima(4)*, *Ocillatoria simplissima (5)*, *Closterium navicular(6)r*, *and Oscillatoria sp.(7)*) were tested for their ability to produce L-asparaginase enzyme.

Seven microalgae (*Oscillatoria nigroriridus*, anabaena sp., *Chroococcus turigidus*,*Oscilatoria acitissima*, *Ocillatoria simplissima*, *Closterium navicular*, and *Oscillatoria sp.*) were tested for their ability to produce L-asparaginase enzyme. The microalgal isolates were allowed to grow on a modified F/2 medium (supplemented with phenol red as an indicator) at a temperature of 30 °C and pH 8 (Fig. 2). The isolate ***Chroococcus turigidus*** was the most potent isolate for the L-asparaginase production by giving the dark pink color. In addition, the nesslerization method confirmed that the isolate ***Chroococcus turigidus*** had the highest activity of L-asparaginase 212.4 U/ml Results showed that L-asparaginase activity increased gradually to the maximum peak after 10th days of incubation, after which, a gradual decrease was reported in the enzyme activity (Fig. 3).

### Anticancer determination

#### Anti-proliferative effect assessment

**Fig. 4:**
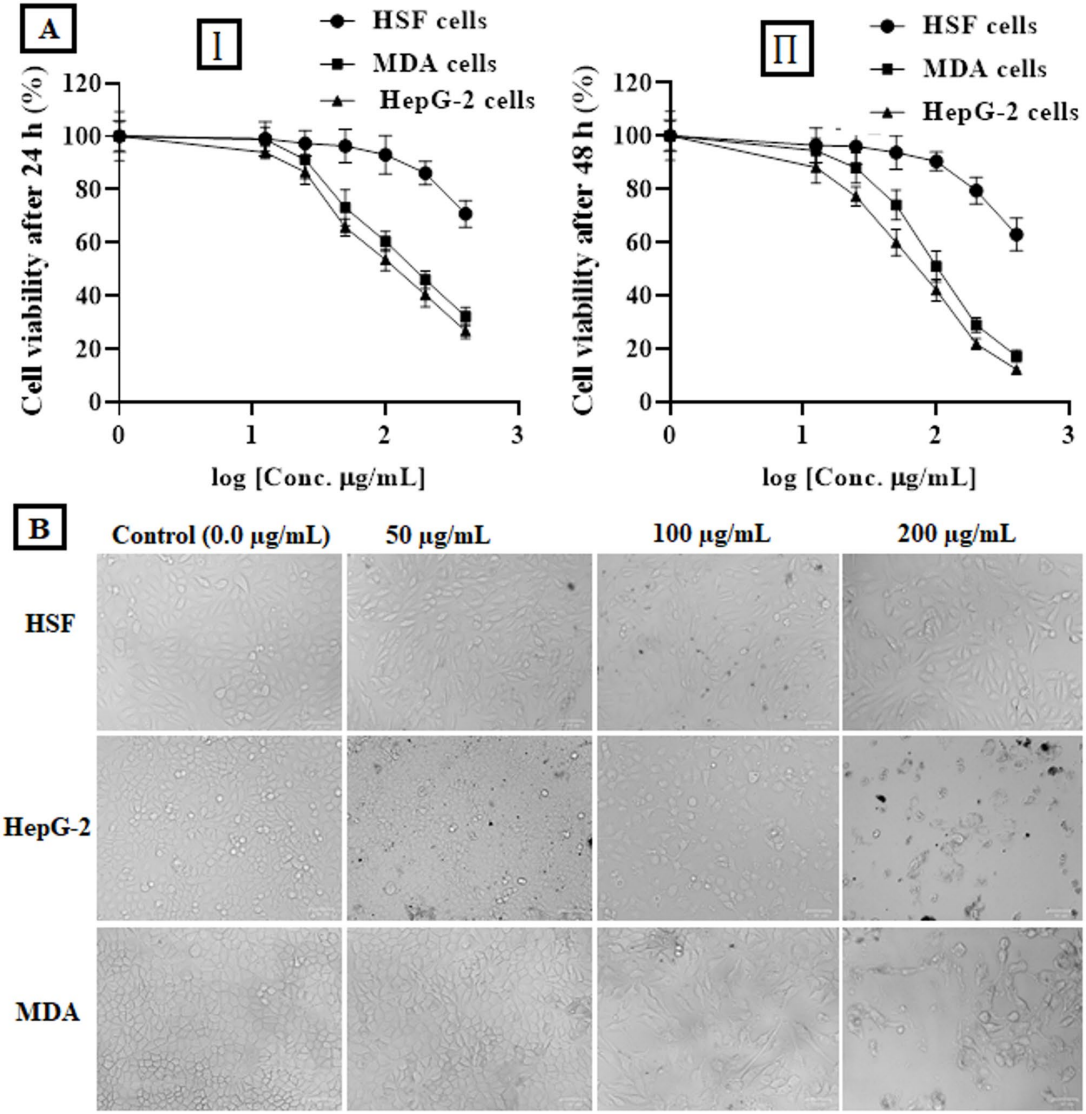
(**A**) Effect of the algal extract on the viability of human skin fibroblasts (HSF), hepatoma cells (HepG-2), and breast carcinoma cells (MDA) at various concentrations (12.5–400 µg/mL) after 24 h (I) and 48 h (II) of treatment compared to untreated controls (mean ± SEM, *n* = 3; *p* < 0.05). (**B**) Representative phase-contrast micrographs showing morphological changes in HSF, HepG-2, and MDA cells following exposure to the algal extract at different concentrations (0, 50, 100, and 200 µg/mL).

The anticancer effect of the algal extract was estimated in vitro against breast carcinoma (MDA) and hepatoma (HepG-2) cell lines in comparison with normal human skin fibroblast (HSF) cell line. The IC_50_ values of the algal extract against HSF cells were Determined to be 844.4 and 730.5 µg/mL after treatment for 24 h and 48 h, respectively (Table 1), representing its high safety profile toward the tested normal cells (Fig. 4AI,AII). Conversely, the algal extract showed a potent effect exerted in a dose-dependent manner against both treated MDA and HepG-2 cancer cells (Fig. 4A). After treatment for 24 h, the algal extract showed low IC_50_ values of 126.3 and 169.8 µg/mL with SI of 6.69 and 4.97, respectively. The obtained findings showed that HepG-2 cells were slightly more sensitive to the treatment than the MDA cells. Furthermore, Table 1 showed that the algal extract exhibited superior anticancer activity against the treated MDA and HepG-2 cells after treatment for 48 h with estimated IC_50_ values of 71.88 and 109.9 µg/mL with SI values of 10.16 and 6.65, respectively. Consequently, morphological changes of the treated HepG-2 and MDA cancer cells were more severe for the algal extract than the normal HSF cells (Fig. 4B).

### Nuclear staining assessment

**Fig. 5:**
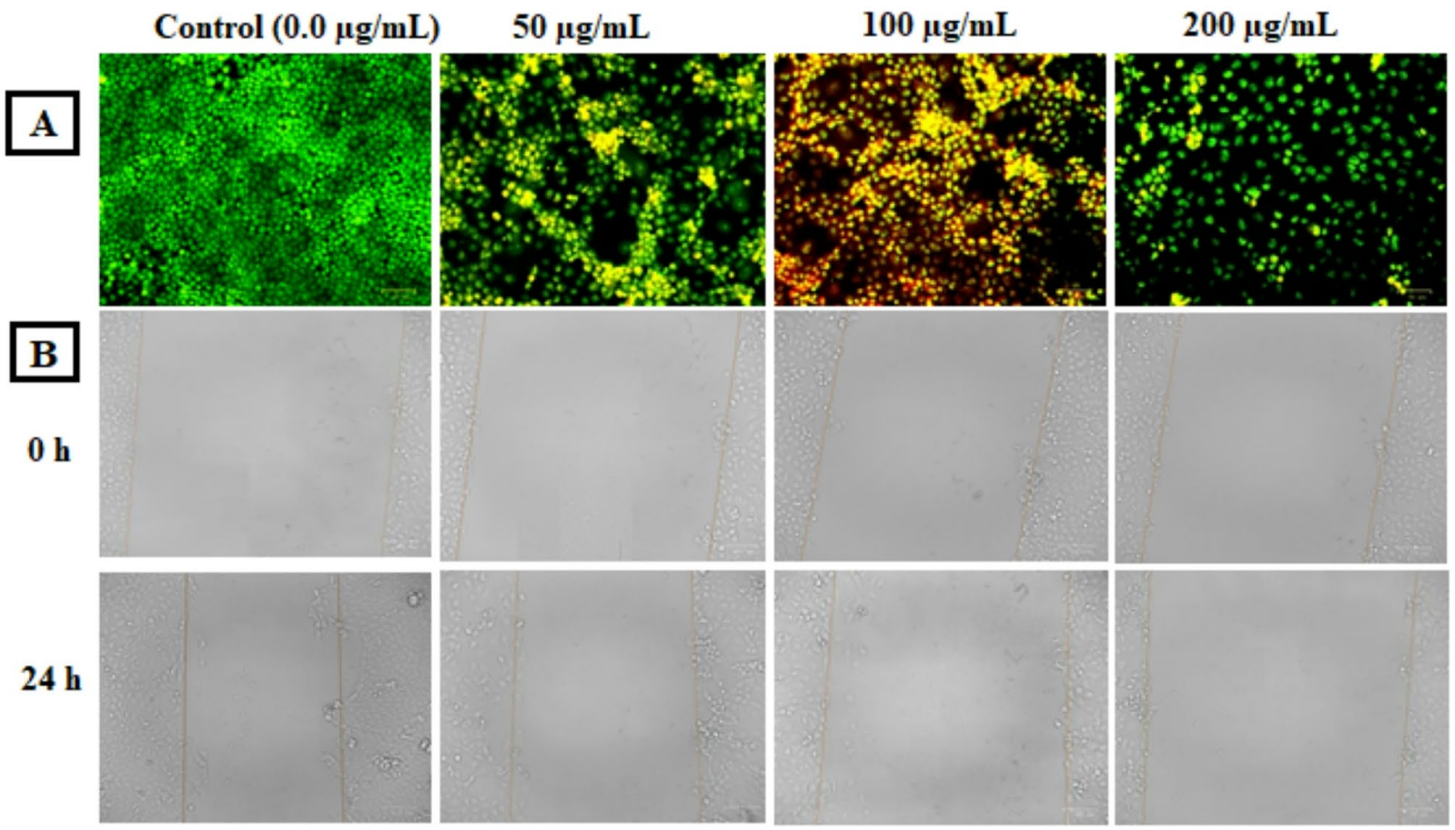
Apoptotic effect of algal extract on the treated cancer cells. (**A**) Fluorescence microscopic images of the treated HepG-2 after staining with acridine orange/ethidium bromide dual stain (Green, yellow, and reddish orange fluorescences indicate healthily viable, early apoptotic, and late apoptotic nuclei, respectively). (**B**) Anti-migration efficacy using wound healing assay via scratched wound area in the untreated and algal extract-treated HepG-2 cells at 0 and 24 h (scale bar 100 μm).

The apoptotic efficacy of the algal extract was confirmed by fluorescence analysis of the treated HepG-2 cells, after staining with dual AO/EB stain, which shows yellow to reddish-orange fluorescence of apoptotic nuclei in comparison with green fluoresce of healthy nuclei of the reference (untreated) cells. The nuclei of the treated-HepG-2 cells Demonstrate yellowish fluorescence at a low concentration of 50 µg/mL, which converts to orange fluorescence after treatment with a concentration of 100 µg/mL, and reddish-orange fluorescence after treatment with a concentration of 200 µg/mL confirming its potent apoptotic activity (Fig. 5A).

### Anti-migration potency assessment

Interestingly, the anti-metastatic activity of the algal extract was Determined using a wound-healing assay. The anti-migration efficacy is Dependent on the inhibition of the activity of migration-related proteases. After treatment for 24 h, the algal extract exhibited high potency to inhibit migration of the treated HepG-2 by 100% compared to untreated control cells, which showed a normal migration property (Fig. 5B).

**Table 1:** The antitumor activity of the algal extract against MDA and HepG-2 cells as compared to normal human cells (HSF) expressed in IC_50_ (µg/mL) and SI values.

Time	Value	HSF	MDA	HepG-2
24 h	IC_50_	844.4 ± 25.45	169.8 ± 10.46	126.3 ± 7.36
	SI	–	4.97 ± 0.15	6.69 ± 0.21
48 h	IC_50_	730.5 ± 17.99	109.9 ± 6.38	71.88 ± 4.17
	SI	–	6.65 ± 0.16	10.16 ± 0.42

## Discussion

The marine environment is a very rich source of different kinds of microorganisms producing many bioactive natural products. L-asparaginase (E.C. 3.5.1.1), is among the relevant enzymes that can be obtained from marine sources. Cyanobacteria and eukaryotic microalgae have great economic and ecological importance. Cyanobacteria, formerly named blue-green algae are the only known prokaryotes capable of oxygenic photosynthesis^23^. They are considered among the oldest life forms on Earth and are the original producers of the Earth’s oxygenic atmosphere^24^.

Seven microalgae were tested for their ability to produce L-asparaginase enzyme. The growth of microalgae increased and reached its maximum value at the stationary phase. Then, started to decrease, in this respect, most studies on the biochemical production of algal and their analysis were carried out in the stationary phase of growth period^25^. In addition, nesslerization reaction was carried out to confirm the production of L-asparaginase. The growth rates of culture and enzyme synthesis are two main characteristics which are mainly influenced by incubation time^26^. The inhibition effect of the algal extract may be related to many migration-related proteins such as matrix metalloproteinase proteins (MMP2 and MMP9)^27^. All photomicrographs represent that the morphology of both MDA and HepG-2 cells were modified in a noticeable manner as well as induction of cell destruction with increasing the treatment doses (from 50 µg/mL to 200 µg/mL). These morphological modifications were combined with the formation of apoptotic body cell shrinkage, cytoplasmic vacuolization, and nuclear condensation with no effect on normally treated HSF cells.

A limitation of this study is that we did not purify the enzyme, and therefore the observed anticancer effects may be influenced by other metabolites present in the crude extract. Future work should focus on enzyme purification, structural characterization, and in vivo validation. Additionally, comparative safety assessments with bacterial L-asparaginases, which are often associated with immunogenicity, are warranted.

## Conclusion

This research demonstrates that the marine cyanobacterium of L-asparaginase enzyme. These findings suggest that this algal could serve as an alternative candidate for applications in pharmaceutical and biotechnological processes.

## Data Availability

The datasets utilized in this research can be found in publicly accessible databases as referenced in the paper citations. However, the data and figures generated throughout this study can be obtained upon request from the corresponding author.
